# Immunity and Tuberculosis

**Published:** 1901-11

**Authors:** 


### Immunity and Tuberculosis.

Dr. H. M. King, in the October 12th number of the *New York Medical Record,* has an article on the influence of heredity on the development of tuberculosis, in which he takes the stand that the children of tuberculous parents possess a certain degree of immunity which renders them less liable to contract the disease than are the children of healthy parents. He bases this conclusion on the study of 242 cases of pulmonary tuberculosis. The results of his observations are summarized as follows: 1st. The percentage of consumptives having tuberculous parentage is actually smaller than that of those having a non-tuberculous parentage, and much smaller than would be more than accounted for by the additional risk of infection to which the former class is subjected. 2nd. Tuberculosis in the parents renders to no inconsiderable extent an immunity to the disease in the offspring, an immunity which of course is but relative, but still demonstrable, as is shown by the increased resistance to the progress of the disease and the increased tendency to recover among this class. R.

